# Interspecific recognition based on cuticular hydrocarbons mediates reproduction control in aphids

**DOI:** 10.1038/s41598-024-54019-7

**Published:** 2024-02-19

**Authors:** Yang Li, Nousheen Parven, Shin-ichi Akimoto

**Affiliations:** 1 Department of Ecology and Systematics, Graduate School of Agriculture, Hokkaido University, Sapporo, 060-8589 Japan; 2 College of Biology and Agriculture, Zunyi Normal University, Zunyi, 563006 Guizhou China; 3 Department of Zoology, University of Dhaka, Dhaka University Campus, Dhaka, 1000 Bangladesh; 4 The Hokkaido University Museum, Hokkaido University, Sapporo, 060-0810 Japan

**Keywords:** Evolutionary ecology, Entomology

## Abstract

The preset study tested whether an aphid species can control its reproduction by recognizing the presence and density of a rival species. *Acyrthosiphon pisum* and *Megoura crassicauda* often coexist on the same leguminous plant. We established clonal colonies from each species and mixed colonies with one *A. pisum* and one *M. crassicauda* adult. There were no significant differences in the population growth patterns of the two species at 20 °C. However, mixed colonies increased faster and attained larger colony sizes than the clonal colonies. Thus, positive interspecific interactions were confirmed. A mixed colony was dominated by the members of a clone that produced a greater number of newborns in the initial stage, irrespective of the species. Thus, we confirmed the priority effect in the interspecific competition. To simulate the priority effect, 15 glass beads coated with the hexane extract of *M. crassicauda* aphids were attached to a cut leaf, to which one *A. pisum* adult was transferred. The presence of the hexane extract of *M. crassicauda* greatly reduced the reproductive rate of *A. pisum* adults*.* We conclude that aphids can control their reproduction by evaluating the relative density of rivals to fellow aphids based on the cuticular hydrocarbons.

## Introduction

Competition is the primary determinant of genetic diversity, biodiversity, and species composition, particularly, in sessile, clonal invertebrates, and plant and microbial communities^1–5^. Previous studies have indicated that clonal invertebrates, plants, and arbuscular mycorrhizal fungi are capable of recognizing self/nonself or related/unrelated neighbors, and controlling competition intensity through altering resource allocations and growth patterns based on allorecognition^6–13^. Plants use allelochemicals exudating from their roots or volatile organic compounds as cues for allorecognition^13–16^. When plants sense the presence of genetically distinct individuals in their vicinity, they invest more resources in their roots to increase their competitive ability^10,17–19^. It has also been reported that plants can control the intensity of competition against allospecific neighbors depending upon whether conspecifics with high relatedness surround them^20^.

Like other clonal invertebrates, aphids (Aphididae, Insecta), which reproduce clonally on host plants, compete with other conspecific or allospecific clones for nutrition and space^21–28^. Previous studies have reported that competition between aphid species is indirect and mediated by deteriorated quality in the shared host plant, natural enemy and ant mutualists^24,29,30^. However, no information has been obtained about how self/nonself recognition is linked with intra- and interspecific competition in aphids. If aphids have the potential of self/nonself recognition, it is predicted that an aphid clone controls its reproduction or propensity to disperse (the percentage of winged adults) long before deteriorated plant quality or high aphid density reduces its reproductive rate.

Circumstantial evidence indicates that aphids can discriminate between clones^31^. Additionally, competition experiments using different clones showed that aphid colonies comprising two clones increased more rapidly than those comprising a single clone of either type^25,32^. These results confirm self/nonself recognition. However, the difficulty in discriminating clones has hindered our understanding of the general pattern of competitive interactions among aphid clones. Our previous study using a color mutant revealed that pea aphids, *Acyrthosiphon pisum* (Harris, 1776)*,* can recognize self/nonself clones and that when a clone is outcompeted in number by a rival clone on the same leaf, it restrains its reproduction, avoiding competition with no chance of winning^32^. Grainger et al.^28^ also reported that between aphid species using the same host plant, the order of aphid arrival on a host plant and ambient temperature determined the outcome of interspecific competition. These studies suggest that the precedence of reproduction by a clone is overwhelmingly advantageous in clone-clone competition in aphids.

Focusing on interspecific competition between *A. pisum* and *Megoura crassicauda* Mordvilko, 1919, the present study firstly tested whether the two species are capable of self/nonself recognition under the condition of low densities and can control their reproductive behavior based on allorecognition. In our previous study, we used only a pair of a color mutant and its original clone for the competition experiment^32^; thus, we did not evaluate whether the result could be applied to aphids in general. Secondly, the present study clarified which cues were used by aphids to discriminate self/nonself clones.

Although some genetic markers have been used to discriminate between aphid clones^25–27,33^, these methods are destructive, hindering the direct observation of interspecific interactions. To overcome the limitation, we used the agar-cut leaf method^34^, which enabled us to count the daily numbers of the two species on the same leaves and understand the dynamic interactions between allospecific clones.

Many insect species commonly use cuticular hydrocarbons (CHCs) to recognize colony members/non-members^35–37^, mates/non-mates^38–43^, and conspecifics/allospecifics^44^. In addition, aphid CHCs are used as a cue when ants choose aphid colonies they should attend to^45^. Therefore, in the present study, we tested whether the cuticular substances of aphids, mainly CHCs, are used as cues for self-recognition. For this test, the cuticular substances of the aphids were extracted using hexane, and small glass beads were coated with the hexane extract. We attached such glass beads to a cut leaf as a dummy for rival aphids. To create a situation where more rival aphids are present on the same leaf, 15 glass beads coated with the hexane extract of *M. crassicauda* aphids were attached on a cut leaf, to which one *A. pisum* adult was transferred to observe its reproductive behavior. Similarly, using glass beads coated with the hexane extract of the fellow clone, we simulated a high density of the fellow clone to evaluate the reproductive rate of an introduced *A. pisum* adult. Thus, the aphid-mimicking experiments compared the effect of high density between the fellow and rival species, with the analysis of the CHC profiles of the two species.

## Results

### Competition and control of reproduction

**Figure 1:**
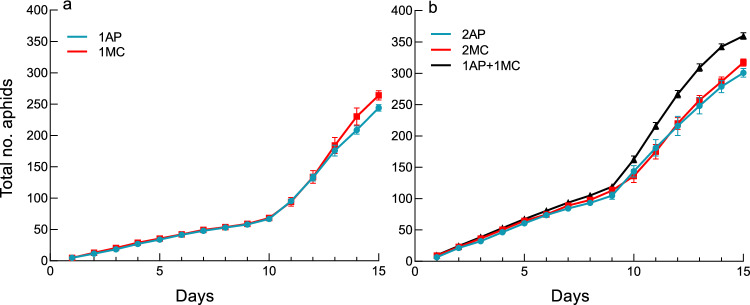
The total daily number of an *A. pisum* (AP) or a *M. crassicauda* (MC) clone at 20 °C. (**a**) The single aphid treatment (1AP or 1MC). (**b**) The two-aphid treatment (2AP or 2MC) and the mixed-clone treatment (1AP + 1MC).

In both species, second-generation aphids produced on day 1 began to reproduce on day 10 at 20 °C, resulting in a steeper population growth curve from day 10 (Fig. 1). There was no significant difference between the two species in the population growth curve in the single-aphid (Fig. 1a; interaction between days and clones, df = 1, *χ*^*2*^ = 1.28, *P* = 0.26) or two-aphid treatments (Fig. 1b; df = 1, *χ*^*2*^ = 0.71, *P* = 0.40). For mixed colonies, significant interspecific interactions were observed; mixed colonies increased more rapidly in total number and attained a greater colony size than the two-aphid colonies of both species (Fig. 1b; for 1AP + 1MC vs. 2AP, the interaction between days and treatments, df = 1, *χ*^*2*^ = 7.83, *P* = 0.0051; for 1AP + 1MC vs. 2MC, df = 1, *χ*^*2*^ = 14.56, *P* = 0.0001). This result confirms positive interspecific interactions.

A density-dependent reduction in reproduction was detected by comparing single-aphid and two-aphid treatments. In *A. pisum* and *M. crassicauda*, the final clone size of the two-aphid treatment was on average 123.1% and 120.2%, respectively, of that of the single-aphid treatment (Fig. 1a, b). These figures were much fewer than 200%, which is expected if the two clonal aphids reproduce without constraints. This result suggests that when two clonal foundresses shared the same arena, they reduced their reproductive rate even if new leaves are supplied continuously.

**Figure 2:**
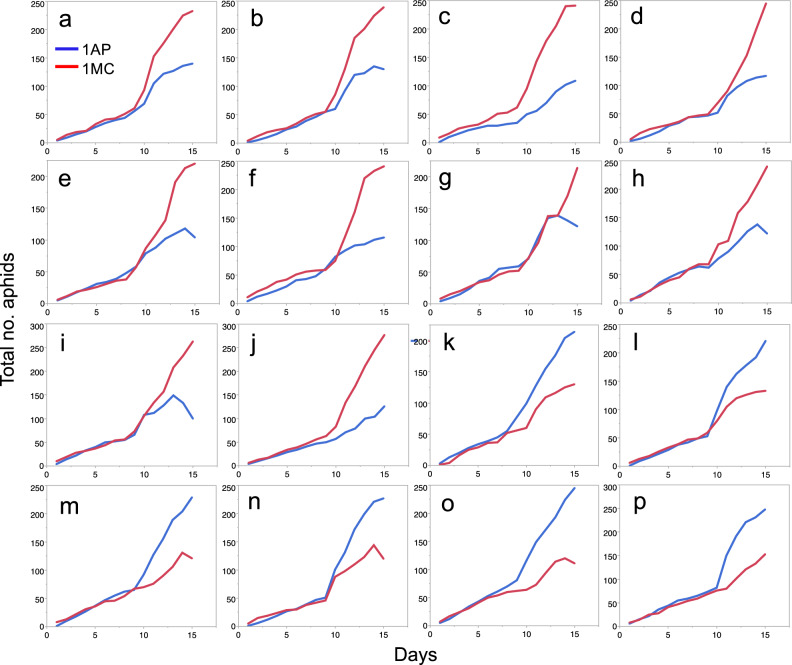
The total daily number of an *A. pisum* (AP) or a *M. crassicauda* (MC) clone in the mixed-clone treatment (1AP + 1MC) at 20 °C. Of the 16 replications, MC won the competition (more than 60% of the entire population) in 10 colonies (from **a**–**j**), while AP won in 6 colonies (from **k**–**p**).

**Figure 3:**
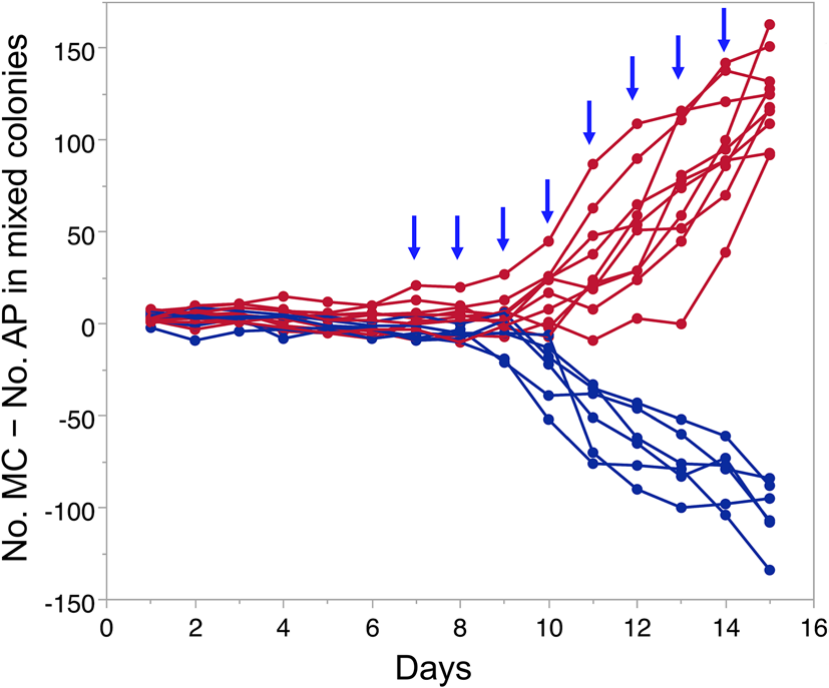
The difference in the total daily number between an *A. pisum* (AP) and a *M. crassicauda* (MC) clone (No. MC—No. AP) for each cage in the mixed-clone treatment (1AP + 1MC) at 20 °C. Red and blue lines indicate cages in which *M. crassicauda* and *A. pisum* won the competition (more than 60% of the entire population), respectively. Arrows indicate significant correlation between the difference on that day and the difference on day 15.

In the mixed colonies, the population growth curves of the two species varied largely between cages (Fig. 2). Although the reproductive rate was almost equal between *A. pisum* and *M. crassicauda* (Fig. 1a, b), there were no cases in which the two species had a similar colony size, but either species always outnumbered the other. Of the 16 mixed colonies reared, *M. crassicauda* accounted for more than 60% of the entire population in 10 colonies (Fig. 2a–j), whereas *A. pisum* accounted for more than 60% in the six colonies (Fig. 2k–p). The species that won the competition (higher than 60%) could be predicted from their earlier colony sizes before the second-generation aphids began to reproduce. Differences in the numbers of *M. crassicauda* and *A. pisum* (no. MC—no. AP) in each cage on day 15 correlated with the differences in the number between the two species as early as day 7 (Fig. 3, Spearman’s rank correlation *ρ* = 0.650, *n* = 16, *P* = 0.0065); this result suggests that a subtle difference in colony size between the two species in early stages led to the acceleration or deceleration of reproduction by the second generation of each species.

**Figure 4:**
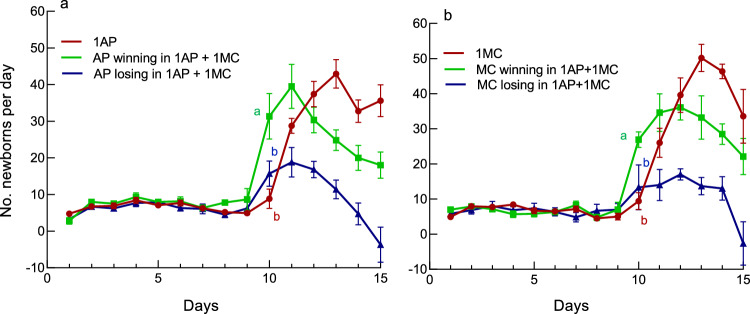
The number of newborns per day (mean ± SE) of an *A. pisum* (AP) or a *M. crassicauda* (MC) clone at 20 °C. (**a**) AP in the single treatment (1AP) and AP that won or lost in the mixed-clone treatment (1AP + 1MC). The numbers of newborns on day 10 were tested using Tukey–Kramer test. (**b**) MC in the single treatment (1MC) and MC that won or lost in the mixed-clone treatment (1AP + 1MC). The numbers of newborns on day 10 were tested using Tukey–Kramer test.

We recorded the daily number of newborns of each species in the mixed colonies when it won (higher than 60%) or lost the competition (smaller than 40%) (Fig. 4a, b). When *A. pisum* won in the mixed colonies*, A. pisum* adults drastically increased the reproductive rate on day 10, when the number of newborns was larger than that in the single-aphid colonies and in *A. pisum* colonies that lost (Fig. 4a, Tukey–Kramer test at the 5% significance level). Similarly, when *M. crassicauda* finally won in the mixed colonies*, M. crassicauda* adults drastically increased the reproductive rate on day 10 to higher than that in the single-aphid colonies and *M. crassicauda* colonies that lost (Fig. 4b, Tukey–Kramer test). For each day from day 11 to day 15, significant difference was found in the daily number of newborns between aphids that won and lost the competition in both species (Fig. 4a, b, ANOVA, for AP, df = 1,14, *F* = 10.00–11.66, *P* < 0.0098; for MC, df = 1,14,* F* = 5.57–15.48, *P* < 0.0333).

In the mixed colonies, the number of winged *A. pisum* adults produced when it won the competition (8.7 ± 2.42 SD) was not significantly different from the number of them when it lost (7.8 ± 3.39 SD) (ANOVA, df = 1,14, *F* = 0.30, *P* = 0.59). Similarly, the number of winged *M. crassicauda* adults produced when it won the competition (14.4 ± 7.96 SD) was not significantly different from the number of them when it lost (18.2 ± 7.23 SD) (ANOVA, df = 1,14, *F* = 0.89, *P* = 0.36).

### Glass beads experiments mimicking rival and fellow aphids

**Figure 5:**
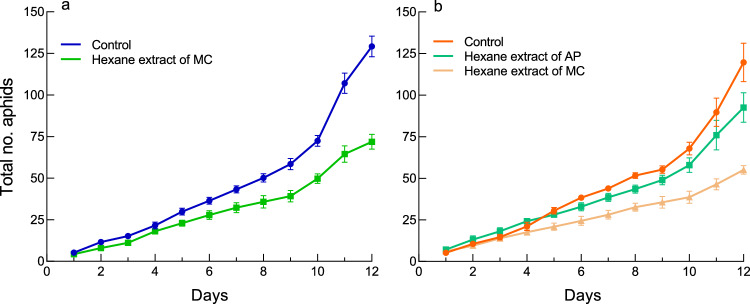
The total daily number (mean ± SE) of an *A. pisum* clone that coexisted with dummy rival aphids, fellow aphids, or control. (**a**) An *A. pisum* clone that coexisted with glass beads coated with the hexane-extracts of a *M. crassicauda* clone or control glass beads. (**b**) An *A. pisum* clone that coexisted with glass beads coated with the hexane-extracts of a *M. crassicauda* clone, the hexane-extracts of the fellow clone, or control glass beads.

When *A. pisum* adults were placed with glass beads coated with the hexane extracts of *M. crassicauda* (648.5 aphids on average), their population growth rates were lower than those of *A. pisum* adults in the control (Fig. 5a; interaction between days and treatments, df = 1, *χ*^*2*^ = 32.11, *P* < 0.0001), with a final colony size of 55.6% of the control. In contrast, when the population growth rate was simultaneously compared among *A. pisum* adults that were placed with glass beads coated with hexane extracts of the same clone members (523.5 aphids on average), glass beads coated with the hexane extracts of *M. crassicauda* (450.8 aphids on average), and control glass beads, the hexane extracts of *M. crassicauda* most strongly restrained the population growth rate of *A. pisum* (for MC extracts vs. AP extracts, df = 1, *χ*^*2*^ = 12.34, *P* = 0.0004; for MC extracts vs. control, df = 1, *χ*^*2*^ = 72.90, *P* < 0.0001), followed by the hexane extracts of the same clone members (for AP extracts vs. control, df = 1, *χ*^*2*^ = 27.28, *P* < 0.0001) (Fig. 5b). The final colony size of *A. pisum* with the hexane extracts of the fellow clone was 77.3% that of the control, whereas that of *A. pisum* with the hexane extracts of *M. crassicauda* was 46.0% that of the control. Thus, *A. pisum* adults responded more sensitively to the density of the rival clone than to that of the fellow clone.

**Figure 6:**
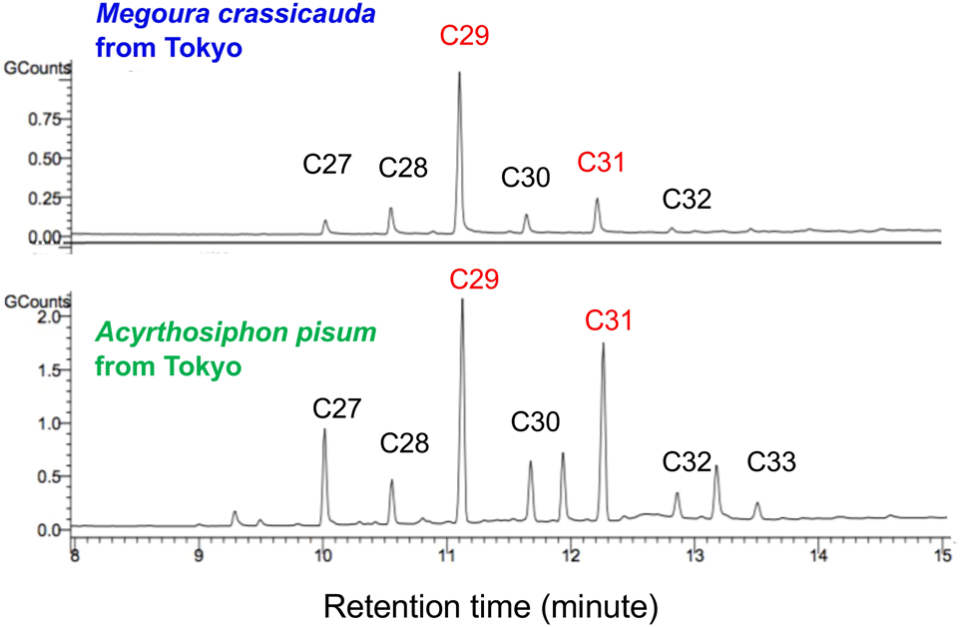
Cuticular hydrocarbon profiles of one *Acyrthosiphon pisum* and one *Megoura crassicauda* clone. The positions of main *n-*alkanes (C27–C33) are indicated. C27, C29, C31, respectively, represent heptacosane, nonacosane, and hentriacontane.

Analysis by GC–MS showed that the two allospecific clones had distinct profiles of cuticular hydrocarbons (Fig. 6). Both species had a mixture of *n*-alkanes, which ranged from *n*-C27 to *n*-C33. Although the amount of *n*-C29 was highest in both species, the relative amounts of cuticular hydrocarbons differed between the species. *A. pisum* had higher amounts of *n*-C27 and *n*-C31 relative to *n*-C29 than did *M. crassicauda*.

## Discussion

The present study successfully described the dynamic process of the competitive interactions between allospecific clones using the agar-cut leaf method. Our results revealed that *A. pisum* and *M. crassicauda* could recognize each other and control their reproductive rates when their population density is low. Because of this ability, the outcomes of the clone-clone competition were one-sided (Fig. 2). The present study also reproduced the outcomes of the interspecific competition by using glass beads to mimic the presence of rival aphids. This is the first study demonstrating that aphids use the cuticular hydrocarbons to recognize the presence of self/nonself clones.

Several studies on interspecific competition in aphids have indicated that the infestation of the host plant by an aphid species has a negative effect on another species that uses the plant later, through the deteriorated quality of the plant or increased natural enemies^22–24,30^. However, it is important to emphasize that reproduction control based on self/nonself recognition occurs much earlier than when the deteriorated quality of the host plant reduces aphid reproductive rates. These results also supported our previous results^32^, corroborating the generality of self/nonself recognition by aphids. Interestingly, aphids decelerated their reproduction when rival aphids outcompeted them but could also accelerate their reproductive rate when they recognized a rival at the initial stage of colony growth (Fig. 4). The presence of rival aphids likely accelerated nymphal growth and promoted reproduction in the initial stage.

The results in Figs. 3 and 4 indicate that the outcome of interspecific competition could vary depending on subtle differences in the initial clone size. Such subtle differences may result from intra-clone variation in reproductive rates and precedence of reproduction by either foundress. Given the aphid’s ability to assess the relative densities of self and nonself clones, subtle differences in initial clone size could be amplified in later stages. This result corresponded with that of our previous study on yellow-green clone competition in *A. pisum*^32^. If the yellow and green foundresses started to reproduce simultaneously, a clone with a higher reproductive rate (green clone) overwhelmed the other (yellow clone). However, in cases where the yellow clone started to reproduce slightly earlier, it overwhelmed the green clone, which restrained its reproduction^32^. Therefore, priority effects^28,46^ are prevalent in the competition between conspecific and allospecific aphid clones.

The aphid-mimicking experiments have indicated that self/nonself recognition of the two species is based on cuticular substances. *A. pisum* adults drastically lowered their reproductive rate when they detected *M. crassicauda* hexane extracts*.* Thus, this result successfully reproduced reproductive restraint in *A. pisum* adults that are outnumbered by *M. crassicauda* aphids in the initial stage. Meanwhile, when *A. pisum* adults sensed the hexane extracts of their fellow aphids, they also reduced their reproductive rate, although the reduction was slight. We found a density-dependent reduction in reproduction when the two-aphid treatment was compared with the single-aphid treatment in both species (Fig. 1a and b). Thus, this density-dependent effect is likely mediated by cuticular hydrocarbons. This result agrees with the observation that in the aphid species *Tuberaphis styraci* producing the solder caste, direct contact among aphids led to the production of solders via non-volatile surface chemicals^47^. Therefore, *A. pisum* adults discriminated between the hexane extracts of rival and fellow aphids, probably by antennation, and responded differently to them.

The reason why an aphid clone restrains reproduction where the rival clone starts reproduction earlier on the same leaf can be explained by considering the explosive potential of aphids to reproduce^48^. The earlier a clone starts reproduction, the lower the possibility that the rival clone can leave its offspring because the earlier-reproducing clone likely occupies the space and nutrition of the host plant, an example of the priority effect^46^. Therefore, when a clone fails to start reproduction earlier, it should produce a high proportion of winged offspring to escape the deteriorating host plant. To maintain a clonal lineage from spring to autumn, aphids must sense the densities of aphid competitors and natural enemies and control their growth and reproduction accordingly. The present experiments did not support the expectation that a clone that loses competition would produce a higher proportion of winged adults. However, to confirm changes in the proportion of winged adults depending on the outcome of the competition, it is necessary to rear nymphs produced after day 10 and analyze their wing morphs.

Clone-clone competition based on self/nonself recognition provides new perspectives on the eco-evolutionary dynamics of aphids. First, intense competition among aphid clones could occur in early spring when several foundresses almost simultaneously start to reproduce on a developing bud or seedling of the host plant^21^. In particular, gall-forming aphids have been reported to compete intensely with conspecific or allospecific foundresses for gall sites and incipient galls^49–54^. Under this competitive condition, foundresses that hatch and start reproduction earlier are more advantageous. Therefore, in aphids, we expect that positive directional selection acts on foundresses to hatch earlier under the same temperature conditions^55^.

In future studies, exploring the range of relatedness aphids recognize as selfness will be necessary. Based on the fact that positive interactions arise when aphids recognize a coexisting clone as nonself, we can understand whether the two clones recognize each other as self. Therefore, by creating mixed colonies consisting of full sibs, unrelated foundresses of the same host race, or foundresses of different host races, we could detect the presence of a competition effect and evaluate the range of selfness.

## Material and methods

### Aphids

One clone each from *A. pisum* (AP) and *M. crassicauda* (MC) was used for all experiments. The clones of the two species were collected from the same clump of *Vicia sativa* ssp. *nigra* on the Tokyo University of Agriculture and Technology (35°41′01′′ N, 139°29′04′′ E) campus, and then maintained monoclonally at 20 °C and a 16L8D photoperiod, using broad bean seedlings^34,56^. Seeds of broad bean were commercially available from Kokusai Petfood (http://www.kpet.co.jp/menu.htm). The collection and maintenance of the aphids and food plant completely comply with national guidelines and legislation and are approved by the Japan Society for the Promotion of Science (19K06848).

To evaluate the population growth patterns of the two species, we transferred fourth-instar aphids onto cut leaves on agar medium containing nutrient solution, allowed them to reproduce, and counted the total number of aphids daily from the first day of larviposition (day 1) to day 15. The aphids were reared in round plastic containers (10 cm in diameter and 5 cm in height) with lids^34^. In the agar-cut leaf-rearing system, aphids grow and reproduce as successfully on cut leaves as they do on broad bean seedlings^34^. While rearing the aphids, we placed the containers upside down to keep the leaf surface clean from honeydew and molted skins. Three treatments were prepared to compare the population growth patterns of *A. pisum* and *M. crassicauda*. First, the single-aphid treatment started with one aphid from each clone (1AP or 1MC) being transferred onto a leaf. Second, the two-aphid treatment was established by transferring two aphids from each pure clone (2AP or 2MC) onto different leaves. This treatment was used to evaluate the density effects of foundresses. Third, in the mixed-clone treatment, one *A. pisum* and one *M. crassicauda* fourth-instar (1AP + 1MC) were simultaneously transferred to different leaves to test whether the coexistence of different species leads to competitive interactions. In each treatment, a freshly cut leaf was added to the unoccupied space of the container every four days without removing old leaves. Aphids moved from old to fresh leaves by themselves, so that aphid colonies were kept undisturbed. A set of three treatments was simultaneously placed under constant temperature conditions of 20 °C using a chamber (NK System, Osaka, LH-200-RDS), which was set to a 16L8D photoperiod at 5.8–7.3 W/m^2^. We prepared 12 replicates for the single-aphid treatment (for each of 1AP and 1MC), 9 for the two-aphid treatment (for each of 2AP and 2MC), and 16 for the mixed clone treatment (1AP + 1MC).

The total number of aphids of each species was counted daily by taking pictures, and the number of newborns per day was estimated by calculating the difference in the total number of consecutive days. The dead aphids were removed and not included in the count. *A. pisum* and *M. crassicauda* were morphologically distinguishable even in the first-instar nymphs; therefore, it was possible to count the aphid number separately for each species in the mixed colonies. In the mixed colonies, the number of winged adults produced during 15 days was counted for each species.

### Glass beads experiments mimicking rival and fellow aphids

To evaluate the effect of the cuticular hydrocarbons of an aphid clone on the reproduction of the rival species and the fellow clone, we extracted cuticular hydrocarbons from *M. crassicauda* and *A. pisum* aphids using hexane. Glass beads were coated with the hexane extracts and were attached to cut leaves, onto which a test *A. pisum* adult was transferred to observe its reproductive activities.

First, we prepared aphids from which the cuticular hydrocarbons were extracted. Four first-instar nymphs of *M. crassicauda* or *A. pisum* were transferred on a broad bean seedling in a cylindrical plastic cage (30 mm diameter and 100 mm height). The aphids were reared until adulthood and were allowed to reproduce for seven days at 20 °C. We prepared four or eight cages simultaneously and used all the aphids, including all instars, to extract the cuticular substances. When eight tubes were prepared, on average, 648.5 (± 38.8 SD, *n* = 15) *M. crassicauda* aphids and 523.5 (± 26.3 SD, *n* = 12) *A. pisum* aphids were used at one time for hexane extraction. When four tubes were prepared, on average, 450.8 (± 8.7 SD, *n* = 12) *M. crassicauda* aphids were used. We transferred all aphids into a 200 mL glass beaker containing a shallow layer of hexane and submerged the aphids for 3 min. All aphids were removed, and 45 glass beads (Toho Co. B-7, green colored, 2 mm in diameter with a hole in the center) were added to the hexane extract and left at room temperature until it completely evaporated.

Of the 45 glass beads coated with hexane extracts, 15 were attached to a cut leaf with wood glue (quick-drying, Konishi), which was then placed on the agar surface (supporting information). An *A. pisum* teneral adult was introduced to the cut leaf and allowed to reproduce. The remaining glass beads were kept at 5 °C, and four days later, 15 of them were attached to a new, freshly cut leaf, which was placed in the container. A new leaf cutting with the remaining 15 glass beads was added to the container four days later. Aphid reproduction was observed daily for 12 days. As a control, we attached the same number of glass beads that were washed only with hexane to a cut leaf to which one *A. pisum* teneral adult was introduced, and, similarly, a fresh leaf cutting with glass beads was added every four days. The experiments using the hexane extracts and the control experiments were paired and conducted simultaneously in the chamber to avoid systematic errors resulting from the experimental periods.

The hexane-extract experiments were conducted in two stages at 20 °C using the same climate chamber (NK System, Osaka, Japan, LH-200-RDS). In autumn 2020, we prepared 12 replicates for the glass bead experiments using hexane extracts from *M. crassicauda* aphids collected from eight cages and 13 replicates for the control experiments. Experiments in spring 2021 utilized 9 replicates for the glass bead experiments using hexane extracts from *A. pisum* aphids collected from eight cages, 10 replicates for the glass bead experiments using hexane extracts from *M. crassicauda* collected from four cages, and 10 replicates for the control experiments.

### Analysis of cuticular hydrocarbon profiles

For the CHC analysis, we used 156 aphids for the *A. pisum* clone and 129 aphids for the *M. crassicauda* clone, including first instars to adults. CHCs were extracted by submerging the aphids in a shallow hexane bath for 60 s. The hexane extracts were analyzed without concentration using the gas chromatography–mass spectrometry (GC–MS) system (Varian/CP-3800 and Varian/1200L, Varian Medical Systems, Inc., Palo Alto, California, USA). The GC–MS system was equipped with the TC-5 column (30 m × 0.25 mm ID, 0.25 μm film; GL Sciences, Shinjuku, Tokyo, Japan). Temperature was kept at 100 °C for 2 min, then increased by 40 °C/min to 200 °C, by 20 °C/min to 260 °C, by 10 °C/min to 305 °C, and finally by 5 °C/min to 325 °C. Helium was used as carrier gas with a constant flow of 1.8 mL/min. Analyses were run in a splitless mode with an injector temperature of 300 °C. Electron ionization mass spectra were recorded with an ionization voltage of 70 eV and an ion source temperature of 250 °C. Components were identified by their characteristic mass spectral fragmentation patterns and retention times.

### Statistical analysis

Population growth curves were statistically compared using generalized linear mixed models (GLMMs) with a Poisson error structure. In the model, the number of aphids on each day was specified as the response variable, whereas days and clones (or treatments) were treated as explanatory variables. We repeatedly counted the number of aphids in each cage so that the numbers were not independent because of time autocorrelation. Additionally, leaf quality and aphid density may vary incidentally among cages. Thus, the differences among the containers were designated as random effects in the GLMM. The month in which the experiment was conducted was added to the model as a block to avoid systematic errors resulting from the experimental period. The glmer function in the “lme4” package in R version 4.0.5^57^ was used for the GLMM. After the results of GLMM were transferred to the Anova function in the “car” package, the significance of the explanatory variables and their interactions was tested using the log-likelihood ratio test (*χ*^*2*^ test). Significant interactions between days and clones (or treatments) were regarded as a statistically significant difference among the population growth curves. Therefore, we have only indicated the results of statistical tests for the interactions between days and clones (or treatments) in the “Results” section.

### Supplementary Information

Supplementary Figure S1.

Supplementary Table S1.

## Data Availability

All the data used in this study are included in Supplementary Information files.
