# Supplementary material for: Interspecific recognition based on cuticular hydrocarbons mediates reproduction control in aphids
Source: Sci Rep. 2024 Feb 19;14:4079. doi: 10.1038/s41598-024-54019-7 (PMC10876990; doi:10.1038/s41598-024-54019-7)
Supplement: Supplementary file 1 — Supplementary Figure S1. [file 41598_2024_54019_MOESM1_ESM.docx]

Supporting information


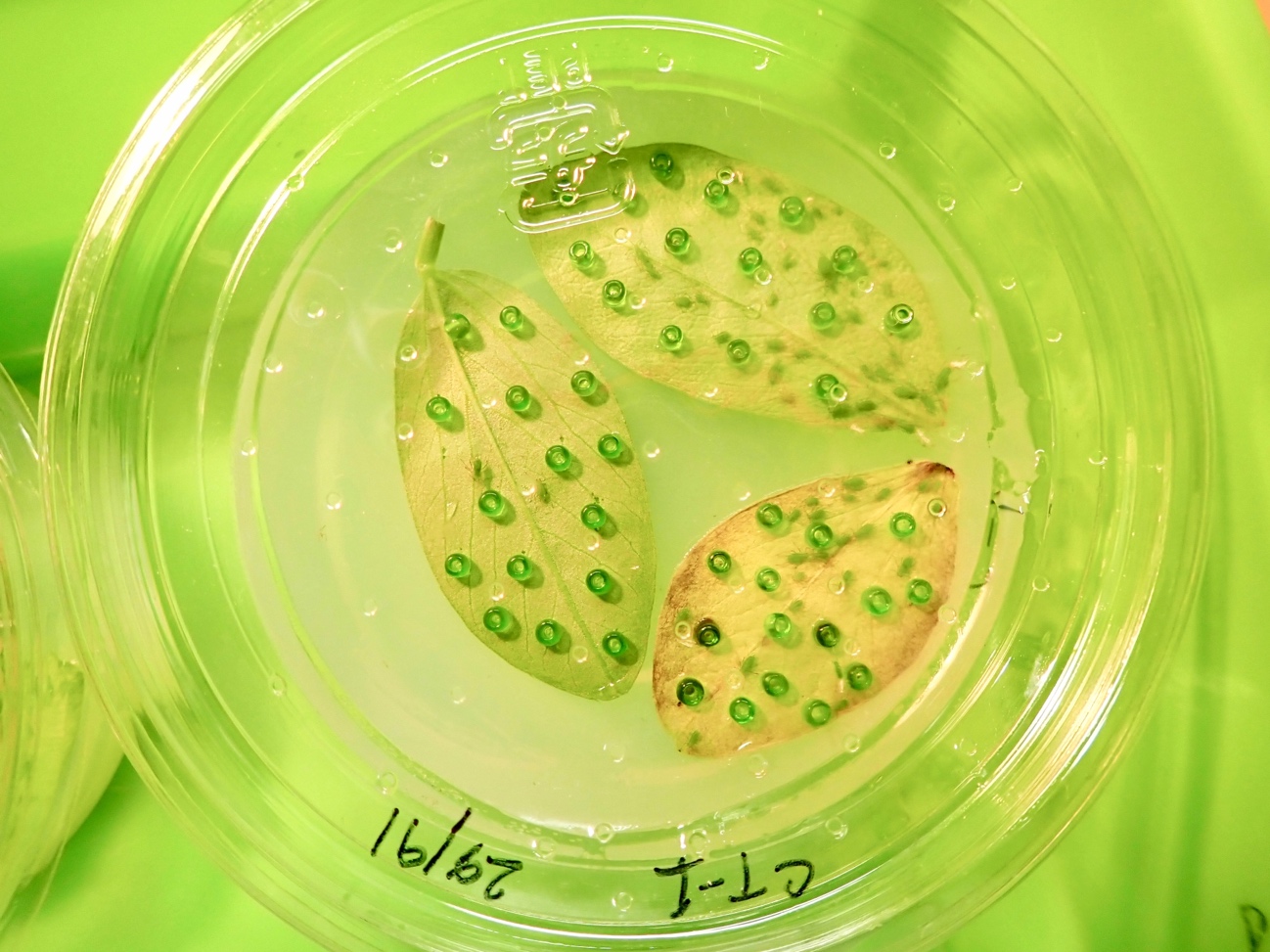
Figure S1. Glass beads experiments mimicking rival and fellow aphids. This picture shows the control experiment. Fifteen glass beads (with or without hexane extract of aphids) were placed on each cut leaf, on which an adult foundress of *Acyrthosiphon pisum* was transferred and admitted to reproduce for 12 days. A cut leaf with 15 glass beads was added on the agar surface every four days.
